# Prenatal diagnosis of dicentric chromosome X mosaicism: a case report and review

**DOI:** 10.3389/fgene.2024.1436469

**Published:** 2024-07-18

**Authors:** Rong Hua Wang, Ke Wu, Xiao Ling Hu

**Affiliations:** ^1^ Department of Laboratory Medicine, Quzhou Maternal and Child Healthcare Hospital, Quzhou, Zhejiang, China; ^2^ Laboratory of Prenatal Diagnosis Center, Quzhou Maternal and Child Healthcare Hospital, Quzhou, Zhejiang, China

**Keywords:** dicentric chromosome, C-banding karyotype, fetal ultrasonography, genetic counseling, chromosomal microarray analysis

## Abstract

A dicentric chromosome is an abnormal chromosome with two centromeres on the same chromosome. It has been reported that dicentric chromosomes are specific biomarkers of radiation exposure, but dicentric chromosomes are rarely identified in newborns with multiple congenital anomalies. At 16 weeks of gestation, a 39-year-old pregnant woman (gravida 2, para 1) was referred to the prenatal diagnosis center for genetic counseling. The fetal ultrasonography indicated multiple anomalies. Subsequently, amniocentesis was performed, and the G-banding karyotype analysis showed a rare type of mosaicism. The C-banding karyotype analysis indicated a pseudo-dicentric chromosome X [psu dic (X; 18) (p11.2; p11.2)]. A single-nucleotide polymorphism array (SNP array) revealed three pathogenic copy number variations (CNVs). After genetic counseling, the parents chose to terminate this pregnancy. This study provides new evidence for a better understanding of the diagnosis of dicentric chromosomes and emphasizes on the importance of genetic counseling.

## Introduction

A dicentric chromosome is an abnormal chromosome with two centromeres on the same chromosome. The occurrence of dicentric chromosomes in cancer cells is a well-recognized event (Mackinnon and Campbell, 2011). It has been reported that dicentric chromosomes are specific biomarkers of radiation exposure (Jeong et al., 2022). However, they are rarely identified in newborns with multiple congenital anomalies. Most dicentric chromosomes are known to form through chromosomal inversions. Dicentric chromosomes mainly (up to 80%) happen between acrocentric chromosomes (Stimpson et al., 2010). Robertsonian translocations (ROBs) involving acrocentric chromosomes (13, 14, 15, 21, and 22) most frequently generate dicentric chromosomes (Chiatante et al., 2017). In this study, we present the first description of a dicentric chromosome (X; 18).

## Materials and methods

**FIGURE 1:**
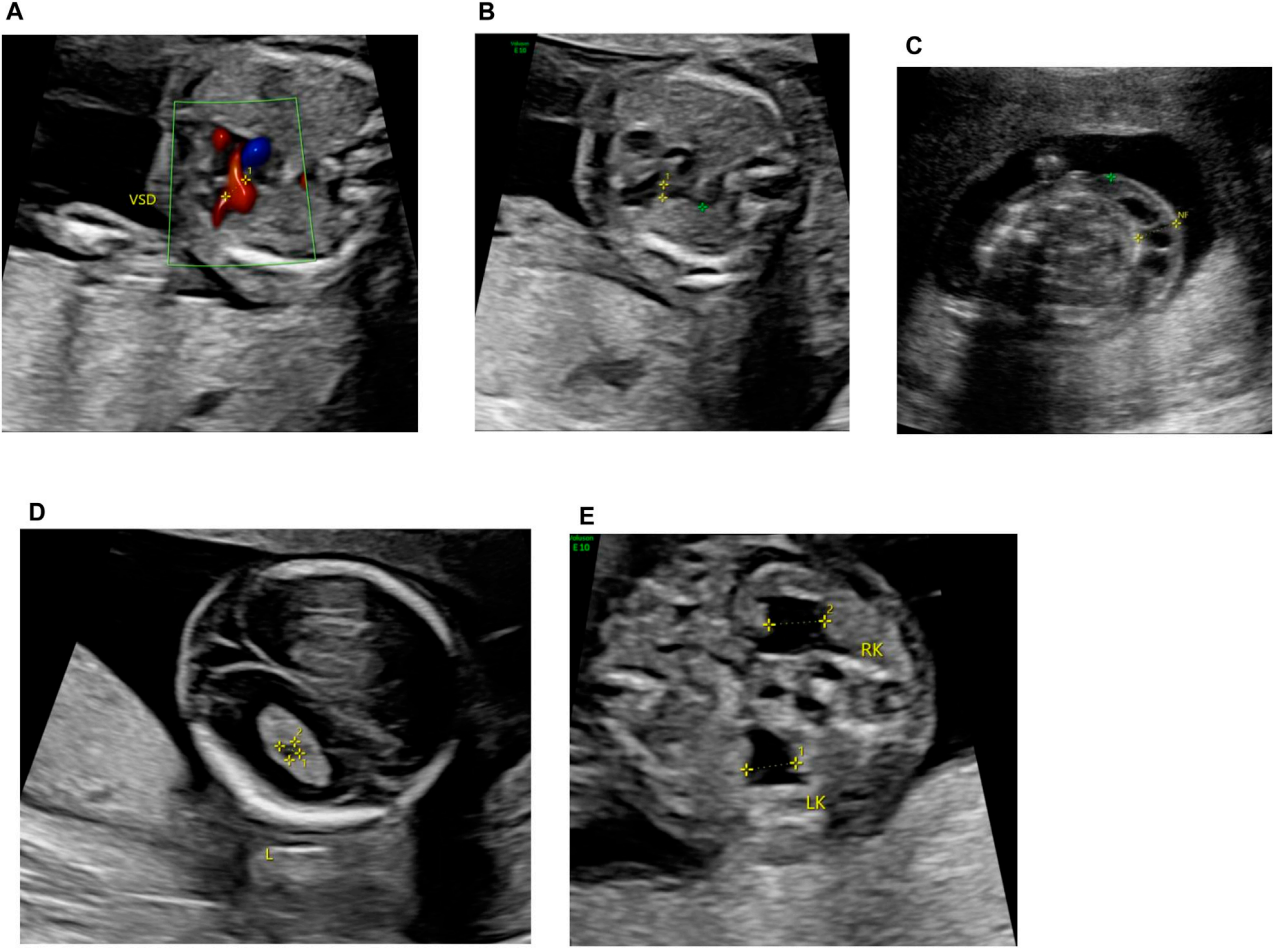
Fetal ultrasonography (at 16 weeks of gestation). **(A)** VSD, **(B)** pulmonary stenosis, **(C)** CCH, **(D)** choroid plexus cyst, and **(E)** bilateral hydronephrosis.

A 39-year-old pregnant woman (gravida 2, para 1) was referred to the center of prenatal diagnosis at Yiwu Maternity and Children Hospital for genetic counseling. At 16 weeks of gestation, the fetal ultrasonography indicated ventricular septal defect (VSD), pulmonary stenosis, cystic hygroma colli (CHC), choroid plexus cyst, and bilateral hydronephrosis (Figure 1).

The parents signed an informed consent for genetic analysis and amniocentesis. Subsequently, the amniocentesis was performed, and the fetal sample detection was performed by single-nucleotide polymorphism array (SNP array) analysis, G-banding karyotype analysis with a band resolution of 400 bands, and C-banding karyotype analysis.

## Results

### G-banding and C-banding karyotype analysis

**FIGURE 2:**
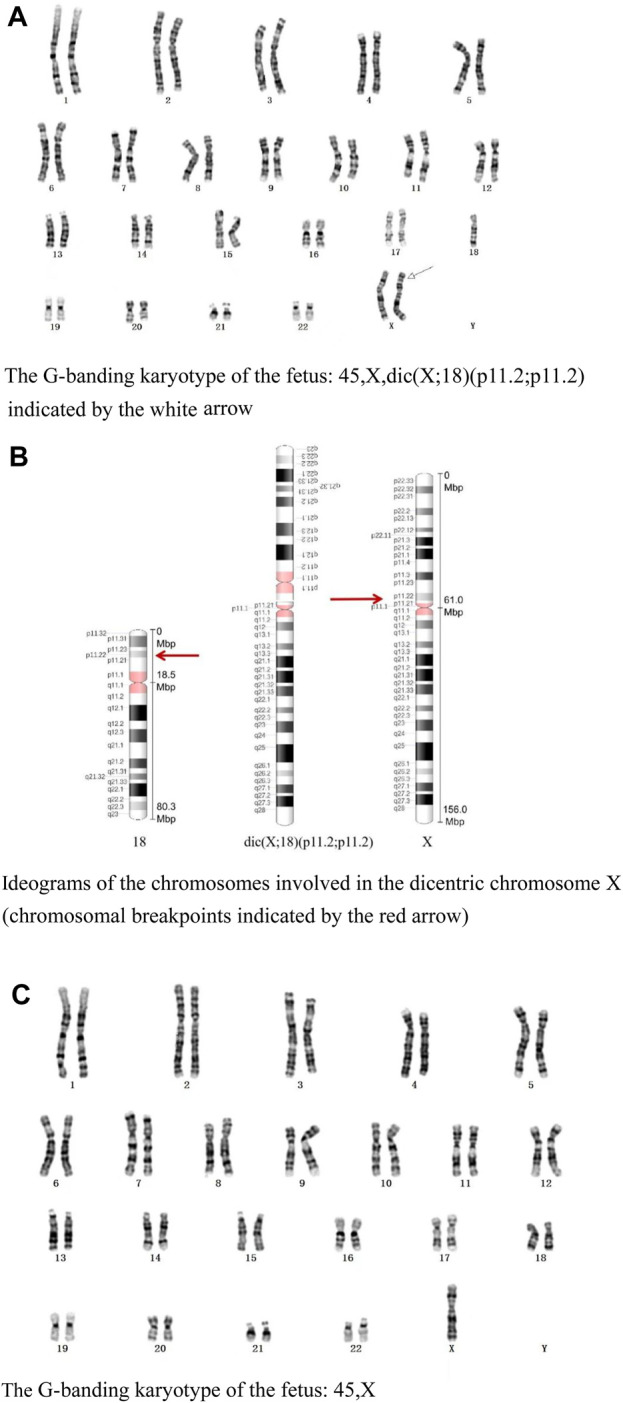
**(A)** G-banding karyotype of the fetus: 45,X,dic (X; 18) (p11.2; p11.2) indicated by the white arrow. **(B)** Ideograms of the chromosomes involved in the dicentric chromosome X (chromosomal breakpoints are indicated by the red arrow). **(C)** G-banding karyotype of the fetus: 45, X.

**FIGURE 3:**
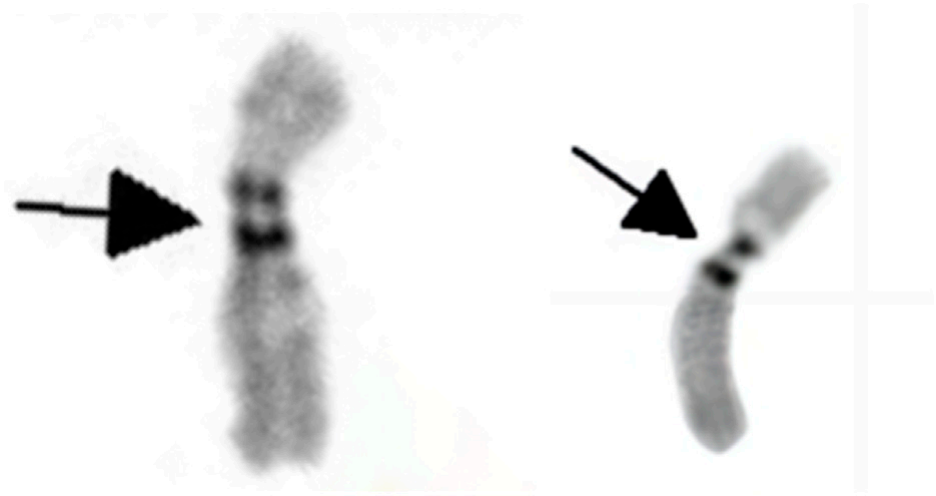
C-banding karyotype of dic (X; 18) (p11.2; p11.2) indicating two centromeres (as shown by the black arrow).

The G-banding karyotype analysis revealed a rare type of mosaicism 45, X, psu dic (X; 18) (p11.2; p11.2) [31]/45,X [26] (Figure 2). The C-banding analysis showed a dicentric chromosome X (Figure 3).

### Chromosomal microarray analysis

**FIGURE 4:**
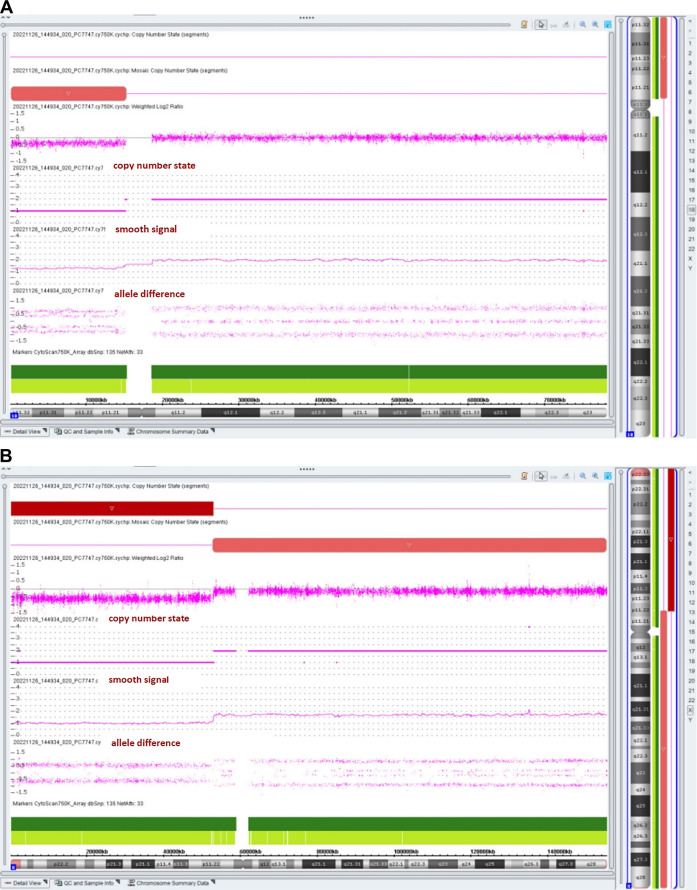
Results of the SNP array analysis (the “smooth signal” representing a copy number, and the “allele difference” with two rows representing haploid regions, with three rows representing diploid regions, and with four rows representing mosaic regions).

The chromosomal microarray analysis (CMA) with an SNP array (Affymetrix CytoScan 750K Array, Santa Clara, California) revealed that the fetus had three pathogenic (Figure 4) CNVs: arr [GRCh37]18p11.32p11.21 (136,228_15181208)×1.32, arr [GRCh37]Xp22.33p11.22 (168,552_52154982) × 1, and arr [GRCh37]Xp11.22q28 (52705315_155233098) × 1.72. The dosage-sensitive genes in CNVs are listed in Table 1.

**TABLE 1:** List of the dosage-sensitive genes in CNVs.

Chromosomal region	Dosage-sensitive genes (ClinGen haploinsufficiency)
18p11.32-p11.21	*TGIF1*
Xp22.33-p11.22	*CDKL5*, *PDHA1*, *RS1*, *ANOS1*, *AP1S2*, *ARSL*, *ARX*, *BCOR*, *CASK*, *CNKSR2*, *DDX3X*, *EBP*, *FANCB*, *GK*, *IL1RAPL1*, *KDM6A*, *MID1*, *NDP*, *NHS*, *NYX*, *OFD1*, *PORCN*, *PQBP1*, *PTCHD1*, *RP2*, *RPS6KA3*, *SLC35A2*, *SMS*, *USP9X*, *WDR45*, OTC, *DMD*, *CLCN5*, *CYBB*, *HCCS*, *NR0B1*, *STS*, *SYN1*, *SHOX*, and *TRAPPC2*

### Pregnancy outcome

Parental karyotypes and CMA were normal. The pregnant woman was informed of these results. After genetic counseling, this family decided to terminate this pregnancy at 20 weeks of gestation.

## Discussion

Chromosome 18p deletion syndrome (OMIM#146390), also known as monosomy 18p, is a rare type of chromosomal syndrome with phenotypic heterogeneity. The clinical manifestations of 18p deletion syndrome include cardiac abnormalities (VSD, pulmonary stenosis, tetralogy of Fallot, and mild aortic valve abnormality), neurologic abnormalities (seizures, hypotonia, and holoprosencephaly), short stature, intellectual disability, holoprosencephaly, hypoplastic pituitary stalk, septo-optic dysplasia, isolated scoliosis, facial dysmorphism, genitourinary abnormalities, gastrointestinal abnormalities, hearing loss, pituitary abnormalities, and ophthalmologic abnormalities (Sebold et al., 2015).

The prevalence is estimated to be about 1:50,000 in live-born infants (Hasi-Zogaj et al., 2015). Over 100 individuals with 18p deletion syndrome have been reported (Kocaaga and Yimenicioglu, 2022), but rare cases (about 10 cases) have been described in prenatal diagnosis. It has been reported that fetuses with a pure 18p deletion present with severe hydronephrosis (Jin et al., 2021), holoprosencephaly (Yin et al., 2017), tetralogy of Fallot (Yi et al., 2014), reduced head circumference (Fogu et al., 2014), increased nuchal translucency (Monosomy, 2009), craniofacial abnormalities, and premaxillary agenesis (Chen et al., 2013). The CMA result of our case showed arr [GRCh37]18p11.32p11.21 (136,228_15181208)×1.32, with a smooth signal of 1.32 and a top allele difference row value of 0.66, indicating that approximately 68% of fetal cells had a 15.0-Mb deletion in the 18p11.32-p11.21 region, which overlapped the 18p deletion syndrome and contained a total of 58 OMIM genes, including a critical dosage-sensitive gene *TGIF1* (OMIM*602630). The heterozygous mutations/deletions of the *TGIF1* gene were associated with autosomal dominant holoprosencephaly 4 (OMIM#142946). This fetus did not show holoprosencephaly. Fetal ultrasonography indicated VSD and bilateral hydronephrosis, which have been reported in literature. The clinical phenotypes and severity may vary with the proportion and distribution of abnormal cells in various tissues.

This case was also a rare variant of Turner syndrome (TS). Vorsanova et al. (2021) reported that Turner syndrome mosaicism occurs in 1.9% of girls with neurodevelopmental disorders and congenital anomalies. The CMA result showed arr [GRCh37]Xp11.22q28 (52705315_155233098)×1.72 (smooth signal was 1.72, and the value of the top allele difference row was 0.86) indicate that approximately 28% of fetal cells had a 102.5-Mb deletion in the Xp11.22-q28 region. The CMA result also showed that the fetus had a 51.9-Mb heterozygous deletion in the Xp22.33-p11.22 region, which contained 264 OMIM genes such as *SHOX* (OMIM*312865). Two copies of the *short-stature homeobox* (*SHOX*) genes are located in the pseudoautosomal region 1 (PAR1) of Xp22.33 and Yp11.3, respectively. *SHOX* plays a particularly important role in short-stature conditions and bone development. *SHOX* has a clear haploinsufficiency effect. SHOX deficiency was associated with Léri–Weill dyschondrosteosis (OMIM#127300) and isolated short stature (OMIM#300582). In the absence of a family history of short stature, SHOX haploinsufficiency-related diseases were rarely diagnosed before late childhood (Ramachandrappa et al., 2018). Li et al. (2023) reported that only 62.5% (5/8) of fetuses with SHOX haploinsufficiency presented with short, long bones (Li et al., 2023). So it is understandable that the ultrasound examination indicated the normal length of the fetal long bones at 20 weeks of gestation.

The conventional C-band karyotype (Zhu et al., 2019) is still an effective complementary method to identify chromosomal heteromorphisms and is also essential for diagnosis of chromosomal disorders. The pseudo dicentric chromosome 45, X, psu dic (X; 18) (p11.2; p11.2) indicated by the C-band karyotype is an unbalanced X-autosome translocation. Some unbalanced X-autosome translocations have been reported to be preferentially inactivated, which could spread X chromosome inactivation (XCI) into the autosomal chromosome regions and inactivate autosomal genes (Favilla et al., 2021). We could not measure the extent of the spreading into the translocated 18. However, the spreading of inactivation into chromosome 18 could not be excluded. In this case, most probably, some genes from the fragment of chromosome 18p11.1-qter attached to chromosome X would be silenced. The spreading of X inactivation into translocated autosomes has been previously observed over large distances.

The *X inactive specific transcript* (*XIST*) gene is located in Xq13.2. *XIST* mediates the X-inactivation center (XIC). It has been found that the deletion of *Xist* is associated with preferential upregulation of XCI escape genes compared to XCI subjective genes, which underlies the variability in *Xist* deficiency phenotypes between different cell lineages and tissues (Yang et al., 2022). So these variable phenotypes may depend on the extent of silenced autosomal genes and genomic imbalances. The fetus was diagnosed with both extremely rare variant Turner syndrome mosaicism and chromosome 18p deletion syndrome mosaicism. The fetus may present with certain phenotypes related to the “silence” of genes in chromosome 18p11.1-qter.

Based on the origin of the centromere, dicentric chromosomes could be categorized into three types (Therman et al., 1986): 1) heterologous dicentric chromosomes derived from non-homologous chromosomes; 2) homologous dicentric chromosomes derived from two homologous chromosomes; and 3) isodicentric chromosomes derived from homologous chromosomes with two identical arms. According to the above categories, this case should belong to type 1, and the derivative dicentric X chromosome is asymmetrical. Individuals with dicentric chromosomes are rarely observed in humans, and we review previously published cases with dic (X; autosomes), which had detailed clinical information (Table 2). They presented with various phenotypes resulting from the extent of the monosomy or trisomy of the concerned segments. Dicentric chromosomes are prone to malsegregation during mitosis, resulting in anaphase bridges and chromosome breakage (Anbumani and Mohankumar, 2015). Most dicentric chromosomes are known to form through chromosomal inversions. Chromosomal inversions are rotations within the chromosomal arm due to chromosomal breakages or intra-chromosomal recombination.

**TABLE 2:** Summary of previously published dic (X; autosomes) cases.

Karyotype	Parent of origin	Phenotype	Reference
45,X,dic (X; 17) (p22.2; p13)	*De novo*	A female newborn was born at the 31st week of gestation, who presented with growth retarded, muscle hypotonia, facial dysmorphism, and multiple dysmorphic features. The girl died of cardiorespiratory distress at the age of 6 days.	Eggermann et al. (1998)
45,X,dic (X; 15) (q26.1; p 11)/45,X	*De novo*	The female newborn presented with global developmental delay, macrocephaly, facial dysmorphism, and multiple dysmorphic features at the age of 3 months. At the age of 23 months, she presented with ataxia and language delay.	Scheuerle et al. (1995)
46,XY, psu dic (X; 15) (p13; p11.1)	Maternal	At the age of 5, the male patient presented with delayed speech and language development, intellectual disability, seizures, and facial dysmorphism	Favilla et al. (2021)
45,X, psu dic (X; 21) (q21.33; p11.1)	*De novo*	The female patient presented with central nervous system abnormalities, atrial septal defect, short stature, hypomimic face, right-hand camptodactyly, feet hypertonia, and hyperhidrosis.	Favilla et al. (2021)
45,X, psu dic (X; 13) (p22.12; q33.3)	*De novo*	The female patient presented with intrauterine growth restriction, neurodevelopmental delay, generalized hypotonia, microcephaly, microsomia, divergent strabismus, facial dysmorphism, short limbs, and urogenital anomalies. The patient died at the age of 2.	Favilla et al. (2021)

As we have seen, the levels of mosaicism performed by conventional karyotype and CMA analyses are different. We know that the procedures for conventional cytogenetic testing and CMA are different. Conventional cytogenetic testing needs a key process of the short-term culture of cells derived from a specimen for generating karyotypes. During the period of the culture, the fetal cells with abnormal karyotypes may not grow well (Hao et al., 2020). So the number of fetal cells with abnormal karyotypes may not be counted. However, the procedures of CMA do not require cultured amniotic fluid cells; they just need direct extraction of cellular DNA (Hao et al., 2020). We consider it an “understandable” discrepancy between the cytogenetic karyotype and CMA results.

In this report, we describe a phenotypically abnormal fetus with mosaic karyotype 45, X, psu dic (X; 18) (p11.2; p11.2)[31]/45,X [26]. This is a rare type of pseudo-dicentric chromosome X that has not been previously reported. Our case report added descriptive information on this fetus and provided a useful reference for genetic counseling.

## Data Availability

The original contributions presented in the study are included in the article/Supplementary Material; further inquiries can be directed to the corresponding author.
